# Tumor Microenvironment Status Predicts the Efficacy of Postoperative Chemotherapy or Radiochemotherapy in Resected Gastric Cancer

**DOI:** 10.3389/fimmu.2020.609337

**Published:** 2021-01-25

**Authors:** Ran Duan, Xiaoqin Li, Dongqiang Zeng, Xiaofeng Chen, Bo Shen, Dongqin Zhu, Liuqing Zhu, Yangyang Yu, Deqiang Wang

**Affiliations:** ^1^ Department of Medical Oncology, Affiliated Hospital of Jiangsu University, Zhenjiang, China; ^2^ Department of Ultrasonography, Affiliated Hospital of Jiangsu University, Zhenjiang, China; ^3^ Department of Oncology, Nanfang Hospital, Southern Medical University, Guangzhou, China; ^4^ Department of Medical Oncology, Jiangsu Province Hospital, Nanjing, China; ^5^ Department of Medical Oncology, The Affiliated Cancer Hospital of Nanjing Medical University, Nanjing, China; ^6^ Nanjing Geneseeq Technology Inc., Nanjing, China; ^7^ The Medical Department, 3D Medicines Inc., Shanghai, China

**Keywords:** tumor microenvironment, chemotherapy, radiochemotherapy, immune infiltration, hypoxia, gastric cancer

## Abstract

**Purpose:**

Chemotherapy (CT) and radiochemotherapy (RCT) are currently the standard postoperative treatments for resected gastric cancer (GC). However, owing to a lack of predictive biomarkers, their efficacy is currently suboptimal. As tumor microenvironment (TME) has the potential to determine treatment response, we investigated the association of TME status with the efficacy of fluoropyrimidine (FU)-based postoperative CT/RCT in resected GC.

**Methods:**

Patients with transcriptome data were screened and selected in three independent cohorts. Favorable (fTME) and poor TME (pTME) were defined by a transcriptome-based TME qualification method. Immune infiltration and hypoxia were assessed.

**Results:**

A total of 535 patients were eligible. fTME, indicating the presence of immune activation, was characterized by NK cell rather than CD8+ T cell infiltration. However, postoperative CT/RCT improved overall survival and disease-free survival time more evidently in patients with pTME GC than those with fTME GC. Stratified by stage in fTME GC, stage III patients benefited from postoperative CT/RCT while stage Ib/II patients did not. In comparison, patients with pTME GC benefited from postoperative CT/RCT, regardless of stage. Furthermore, fTME was more hypoxic than pTME, accompanied by a stronger expression of thymidylate synthase (TS)—the target of FU. Stage Ib/II fTME GC was the most hypoxic and had the strongest TS expression across all the subgroups stratified by TME status and stage.

**Conclusions:**

We found that fTME, with the enrichment of NK cells, may predict the lack of postoperative CT/RCT efficacy in stage Ib/II GC, which may be associated with hypoxia and TS expression. Further validations and mechanism researches are needed.

## Introduction

Gastric cancer (GC) is characterized by high relapse rates even after curative surgery. Two different postoperative therapeutic strategies—fluoropyrimidine (FU)-based chemotherapy (CT) and radiochemotherapy (RCT)—have been verified for use in improving the curative and survival rates in patients with resected GC (1). Both CT and RCT exert equivalent effects in improving patients’ overall survival (OS), while RCT yields superior local control than CT, correlating to better disease-free survival (DFS), especially in stage III GC (2–4). However, regardless of the treatment type, many patients still experience relapse after surgery, independently of the disease stage, suggesting the presence of molecular heterogeneity, in terms of therapy response, among patients.

Currently, there are no validated prognostic or predictive biomarkers for GC patients who receive postoperative CT/RCT. However, recent findings on the molecular mechanisms of GC, obtained using high-throughput methods, may allow for the identification of novel biomarkers. The Cancer Genome Atlas (TCGA) and Asian Cancer Research Group (ACRG) projects—landmarks in the molecular characterization of GC—provide invaluable resources for the development of more comprehensive methods to interpret and combat GC (5, 6).

A large body of evidence, based on the TCGA and ACRG datasets and other Next Generation Sequencing (NGS) data, has recently been obtained. However, few distinct biological targets are found on the tumor cell alone. An increasing amount of attention is now being directed to the tumor microenvironment (TME), considering the significance of tumor-related structures as well as the interaction between tumor cells and other cells in the TME. The clinical relevance of TME-associated biomarkers, which reveal changes in the compositions of resident cell types within the TME during cancer evolution, has been reported for various malignancies (7, 8).

In the current era of widespread immunotherapy use, CT and RCT are still the current cornerstones of GC treatment, especially in the postoperative setting. Gaining further insights into TME may help improve the efficacy of not only immunotherapy but also CT/RCT. A previous study found that cancer-associated fibroblasts, among the immunosuppressive cell types in the TME, can promote irradiated cancer cell recovery and cause radioresistance (9). Another study reported that intratumoral interleukin-17-producing cell infiltration improved the response to CT in GC (10). However, there is still a lack of qualification method for a comprehensive evaluation of the TME status to aid in the prediction of CT/RCT efficacy in GC.

Recently, based on a comprehensive landscape of the TME-associated transcriptome characteristics in ACRG, a methodology for the quantification of TME status—the TMEscore—was established specifically for GC. The TMEscore has been validated as a robust prognostic biomarker in GC (11). However, the association between TMEscore and immune infiltration needs further experimental validation and it remains unknown whether TMEscore is predictive for postoperative CT/RCT efficacy of GC.

Accordingly, in this study we aimed to use the novel TMEscore to investigate the association between TME status and postoperative CT/RCT efficacy in resected GC.

## Materials and Methods

### Patients

This study included three independent cohorts. Patients who were retrospectively screened from GC cases received gastrectomy at the Affiliated Hospital of Jiangsu University (AHJU) composed the AHJU cohort. Informed consent was obtained from all patients and the research protocol was approved by the hospital ethics committee (Grant No: 201750). GC cohorts from the ACRG and TCGA were also analyzed. The enrollment criteria for all patients included: 1) a prior history of gastrectomy; 2) histologically confirmed stages IB–III (≥T2 and/or node-positive and M0) adenocarcinoma of the stomach; 3) available gene expression data for TME status estimation; and 4) a definite treatment history of surgery plus FU-based postoperative CT/RCT or surgery alone. American Joint Committee on Cancer criteria were used for clinical and clinicopathologic classification and staging.

### Transcriptome Data

We used the dataset—EGAD00001004164—from the European Genome-phenome Archive, including 34 consecutive patients who underwent GC surgery in 2016 at the AHJU. In brief, total RNA from fresh samples was extracted using Trizol (Invitrogen, USA). Ribosomal RNA was depleted using RNase H followed by library preparation using KAPA Stranded RNA-seq Kit with RiboErase (KAPA Biosystems, USA). The library concentration was determined using KAPA Library Quantification Kit (KAPA Biosystems, USA), and library quality was assessed by the Agilent High Sensitivity DNA kit on Bioanalyzer 2100 (Agilent Technologies, USA), which was then sequenced on Illumina HiSeq4000 NGS platforms (Illumina, USA). Base calling was performed on bcl2fastq v2.16.0.10 (Illumina, USA) for the generation of sequence reads in the FASTQ format (Illumina 1.8+ encoding). Quality control was performed with Trimmomatic (version 0.33). STAR (version 2.5.3a) was used for transcriptome mapping followed by isoform and gene level quantification, as performed using RSEM (version 1.3.0). ACRG and TCGA GC RNA abundance data were downloaded from the NCBI Gene Expression Omnibus (GSE62254) and University of California Santa Cruz (UCSC) Xena platform (https://xenabrowser.net/datapages/), respectively.

### Tumor Microenvironment Assessment

The TMEscore was estimated using principal component analysis (PCA) based on transcriptome data of genes associated with different TME phenotypes and prognoses in GC, as previously described (11), using the R package available in GitHub (https://github.com/DongqiangZeng0808/TMEscore). In brief, the TMEscore construction included: 1) the TME phenotype was determined by transcriptome-based cell abundance deconvolution, unsupervised clustering and consensus clustering; 2) differentially expressed genes associated with TME phenotypes were identified and TME gene signatures were generated after dimension reduction; 3) principal component 1 from PCA was extracted to serve as the score of TME gene signatures; 4) TMEscore was calculated using the signature score whose Cox coefficient for prognosis was positive to subtract the signature score whose Cox coefficient for prognosis was negative. We defined favorable TME (fTME) and poor TME (pTME) based on the contrast immune infiltration status, using the median TMEscore value as a cut-off.

### Microsatellite Instability Assay

Genomic DNA was extracted from GC and normal tissues, following which single fluorescent multiplex polymerase chain reaction was performed for the detection of five well-known mononucleotide repeats. Further details on MSI and microsatellite stability (MSS) assay as well as their definitions have been provided elsewhere (6).

### RNA-Based Immune Infiltration Quantification and Hypoxia Scoring

Cell, an RNA-based silico tool (12), was used for the quantification of the proportions of the pertinent phenotypes of human immune cells in GC samples. Parametric Gene Set Enrichment Analysis (13), which determines the misregulation of defined gene signatures, was used for the quantification of the degree of tumor hypoxia, based on well-established signatures (14–19).

### Immunohistochemistry

Programmed death ligand-1 (PD-L1) staining using Dako PD-L1 IHC 22C3 pharmDx kit (Agilent Technologies) was performed on a Dako Autostainer Link 48 system (Agilent Technologies), and the specimen was then counterstained with hematoxylin and coverslipped, according to the manufacturer’s instructions. The PD-L1 expression level was determined using the combined positive score (CPS), which is the percentage of PD-L1-positive cells (tumor cells, lymphocytes, and macrophages) relative to all the viable tumor cells present in the sample, multiplied by 100. PD-L1-positivity was confirmed at a CPS ≥1 (20). Anti-thymidylate synthetase (TS, ab108995, Abcam, UK) and anti-ERO1A (ab177156) antibodies were used in a two-step IHC protocol. The presence of nuclear and/or cytoplasm tumor cell staining was considered as positive TS expression, irrespective of the proportion or intensity (21). An IHC score ≥8 (median value) was used to define positive ERO1A expression.

### Multiplexed Immunohistochemistry and Multispectral Imaging

Immune cell subsets in the TME were identified by mIHC and multispectral imaging. Multiplex immunofluorescence staining was performed using PANO 7-plex IHC kit (Panovue, Beijing, China), according to the manufacturer’s instructions. T cells were identified using the CD8 marker. NK cells were identified using the CD56 marker and were divided into two categories according to the intensity of membrane staining for the CD56 protein: CD56dim (weak staining) and CD56bright (strong staining). TAMs were identified by CD68 and HLA-DR and were divided into two categories: type M1 (CD68+ and HLA-DR+) and type M2 (CD68+ and HLA-DR−). Different primary antibodies were sequentially applied, including anti-CD8 (CST70306, Cell Signaling Technology, USA), anti-CD56 (CST3576), anti-panCK (CST4545), anti-CD68 (BX50031, Biolynx, China), anti-HLA-DR (ab92511), and anti-S100 (ab52642). S100 staining was used to define the invasive margin and tumor parenchyma (22). The Mantra System (PerkinElmer, Waltham, Massachusetts, US) was used to scan the stained slides and subsequently build a single stack image. The inForm image analysis software (PerkinElmer, Waltham, Massachusetts, US) was used for the reconstruction of images of sections with autofluorescence removal, based on a spectral library for multispectral unmixing.

### Statistical Analysis

First, we used multiple imputation to generate complete datasets for the subsequent analyses in the ACRG, TCGA, and pooled cohorts that included all patients (**Supplementary Data**), according to the multiple imputation guideline (23). Chi-square tests, Fisher’s exact probability tests, Student’s t tests, Wilcoxon tests, and Mann–Whitney U tests were used for between-group comparisons, as needed. Spearman’s correlation was used for pairs of continuous variables. Survival analyses were performed using Kaplan–Meier plots and log-rank tests. Cox proportional hazards regression analysis was used for the evaluation of prognostic factors and calculation of hazard ratios (HRs) along with their 95% confidence intervals (CIs). The HR for each imputed dataset was estimated, and all the estimated HRs were then combined according to Rubin’s rules (24). The imputed dataset with the closest HR to the combined HR was selected for survival curve plotting. We used 5% as the significance level for all tests. SPSS (version 19.0, Chicago, IL), R (version 3.6.1), and R Bioconductor packages were used for all the above-mentioned analyses.

## Results

### Patient Characteristics

A total of 34, 271, and 230 eligible patients were enrolled from the AHJU, ACRG, and TCGA cohorts, respectively (**Table 1**). MSI GC was more frequently associated with fTME than MSS GC, while about 40% of the MSS cases also showed fTME in each cohort. In terms of the associations between TME status and the other characteristics, differing results were observed across the cohorts, indicating the presence of heterogeneity.

**Table 1:** Patient characteristics according to tumor microenvironment status.

Characteristic^*^	AHJU cohort (%)			ACRG cohort (%)			TCGA cohort (%)		
	pTME	fTME	*P* value	pTME	fTME	*P* value	pTME	fTME	*P* value
Age (years)									
<65	9 (69.2)	4 (30.8)	0.078	76 (52.4)	69 (47.6)	0.359	49 (51.0)	47 (49.0)	0.789
≥65	8 (38.1)	13 (61.9)		59 (46.8)	67 (53.2)		66 (49.3)	68 (50.7)	
Sex									
Female	9 (69.2)	4 (30.8)	0.078	44 (41.1)	42 (58.9)	0.762	41 (45.1)	50 (54.9)	0.225
Male	8 (38.1)	13 (61.9)		91 (49.2)	94 (50.8)		74 (53.2)	65 (46.8)	
Tumor location									
Non-antrum	11 (47.8)	12 (52.2)	0.714	69 (54.3)	58 (45.7)	0.180	58 (43.0)	77 (57.0)	0.011
Antrum	6 (54.5)	5 (45.5)		66 (46.2)	77 (53.8)		57 (60.0)	38 (40.0)	
Histology grade									
I/II	8 (53.3)	7 (46.7)	0.730	47 (38.5)	75 (61.5)	0.001	41 (45.6)	49 (54.4)	0.280
III	9 (47.4)	10 (52.6)		88 (59.1)	61 (40.9)		74 (52.9)	66 (47.1)	
TNM stage									
IB/II	8 (44.4)	10 (55.6)	0.492	45 (35.7)	81 (64.3)	<0.001	64 (48.1)	69 (51.9)	0.504
III	9 (56.2)	7 (43.8)		90 (62.1)	55 (37.9)		51 (52.6)	46 (47.4)	
MSI status									
MSS	15 (53.6)	13 (46.4)	0.368	126 (61.5)	79 (38.5)	<0.001	103 (57.5)	76 (42.5)	<0.001
MSI	2 (33.3)	4 (66.7)		9 (13.6)	57 (86.4)		12 (23.5)	39 (76.5)	
Postoperative chemotherapy or radiochemotherapy									
Untreated	5 (31.2)	11 (68.8)	0.039	55 (49.5)	56 (50.5)	0.942	77 (53.5)	67 (46.5)	0.173
Treated	12 (66.7)	6 (33.3)		80 (50.0)	80 (50.0)		38 (44.2)	48 (55.8)	

*Unimputed data were shown.

AHJU, Affiliated Hospital of Jiangsu University; TCGA, The Cancer Genome Atlas; ACRG, Asian Cancer Research Group; MSS, microsatellite stability; MSI, microsatellite instability.

### Tumor Microenvironment Status and Immune Infiltration Characteristics

**Figure 1:**
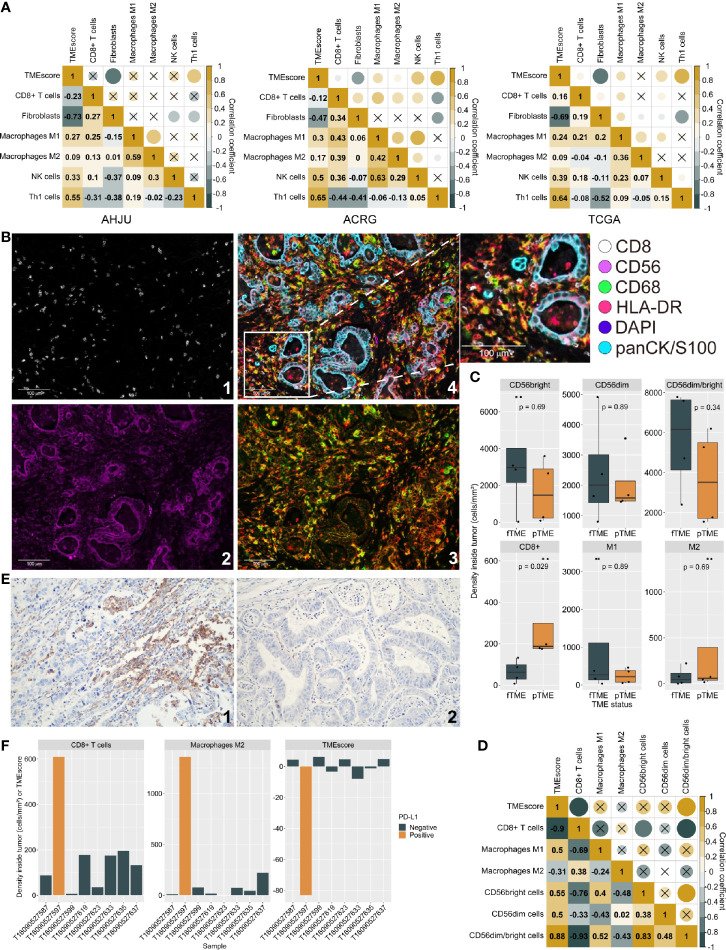
Tumor microenvironment (TME) status and immune infiltration characteristics. **(A)**: Correlation between TMEscore and immune cell abundance based on transcriptome. **(B)**: Typical micrographs of multiplexed IHC and multispectral imaging, at 200× magnification; 1: CD8+; 2: CD56+; 3: CD68+ (green) and HLA-DR (red); 4. Reconstructed image after autofluorescence removal. **(C)** Cell density inside tumor by TME status. **(D)**: The correlation between TMEscore and the effective infiltration degree of varied immune cells. **(E)**: Typical micrographs of programmed death ligand-1 (PD-L1)-positive (1) and negative (2) tumors, at 200× magnification. **(F)**: TMEscore and densities of CD8+ T cells and macrophages M2 inside tumors by PD-L1 expression. AHJU: Affiliated Hospital of Jiangsu University; ACRG, Asian Cancer Research Group; TCGA, The Cancer Genome Atlas; fTME or pTME, favorable or poor TME.

At first, we investigated TMEscore-associated immune infiltration characteristics which need further validation. It is well-known that Th1 cells activate antitumor immunity while fibroblasts suppress it (25, 26). Through transcriptome-based cell type enrichment analysis, we found that the TMEscore positively correlated with the abundance of Th1 cells but negatively correlated with the abundance of fibroblasts in all the cohorts (**Figure 1A**). Moreover, the TMEscore positively correlated with the abundance of NK cells and macrophages M1 in all the cohorts, but negatively correlated with the abundance of CD8+ T cells in the AHJU and ACRG cohorts (**Figure 1A**).

mIHC staining was used to validate the infiltration of selected cells in eight of the AHJU cohort patients that had a sufficient amount of tissue (**Figure 1B**). Compared to pTME GC, fTME GC was associated with higher densities of CD56bright/dim NK cells and macrophages M1 but lower densities of CD8+ T cells and macrophages M2 inside the tumors, despite the significance of these differences were limited by the small sample size (**Figure 1C**). As the mobilization of immune cells from the stromal tumor edge into the tumor parenchyma is crucial for antitumor immunity (22), we next applied the effective infiltration degree (EID: the number of immune cells in tumor parenchyma divided by the total number of immune cells at the stromal tumor edge and in the tumor parenchyma, multiplied by 100%) for the evaluation of antitumor immunity dynamism. We found that TMEscore positively correlated with the EID of CD56bright/dim NK cells (Spearman r = 0.88, *P* = 0.007) but negatively correlated with the EID of CD8+ T cells (Spearman r = -0.90, *P* = 0.005; **Figure 1D**). These results confirmed our transcriptome-based findings.

PD-L1 staining (**Figure 1E**) showed that the only one PD-L1-positive tumor (CPS = 2) from the eight samples had the lowest TMEscore but the highest densities of CD8+ T cells and immunosuppressive macrophages M2 inside the tumors (**Figure 1F**), indicating that the CD8+ T cells inside tumors may be deactivated by PD-L1 signaling and that the TMEscore is a strong indicator of antitumor immunity dynamism.

### Tumor Microenvironment Status and Postoperative Chemotherapy/Radiochemotherapy Efficacy

**Figure 2:**
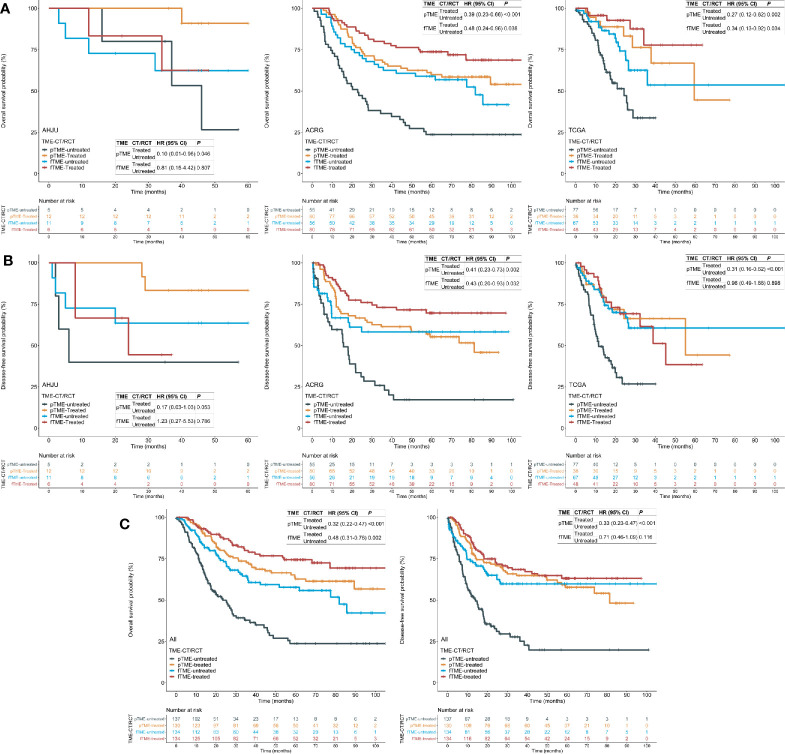
Tumor microenvironment (TME) status and the efficacy of postoperative chemotherapy (CT) or radiochemotherapy (RCT). **(A)** Overall survival (OS) in each cohort; **(B)** disease-free survival (DFS) in each cohort; **(C)** OS and DFS in the pooled cohort. AHJU, Affiliated Hospital of Jiangsu University; ACRG, Asian Cancer Research Group; TCGA, The Cancer Genome Atlas; HR, hazard ratio; CI, confidence interval; fTME or pTME, favorable or poor TME.

First, we confirmed that postoperative CT/RCT improved the OS and DFS of patients with resected GC compared to surgery alone, across all the cohorts (**Figure S1**). Then, we showed that the benefit of postoperative CT/RCT was more pronounced in cases with pTME, in terms of both OS (**Figure 2A**) and DFS (**Figure 2B**). In comparison, these benefits were reduced or even disappeared in cases with fTME (**Figures 2A, B**). These findings were further confirmed in the combined cohort with all patients (**Figure 2C**). Moreover, after univariate selection for the prognostic significance of variables, multivariate models were created. Because a requirement of large sample size, this analysis was conducted in the combined cohort. We showed that postoperative CT/RCT was an independent predictor of both OS (HR = 0.32, 0.21–0.48, *P* < 0.001) and DFS (HR = 0.31, 0.21–0.47, *P* < 0.001) in pTME GC (**Table 2**).

**Table 2:** Univariate and multivariate analyses of variables associated with overall survival and disease-free survival in patients with resected gastric cancer who had poor tumor microenvironment status in the pooled cohort.

Variable	Univariate analysis				Multivariate analysis^*^			
	Overall survival		Disease-free survival		Overall survival		Disease-free survival	
	HR (95% CI)	*P*	HR (95% CI)	*P*	HR (95% CI)	*P*	HR (95% CI)	*P*
Age (≥65 *vs <*65 years)	1.92 (1.33–2.76)	<0.001	1.58 (1.12–2.23)	0.010	1.76 (1.19–2.60)	0.005	1.22 (0.85–1.77)	0.279
Sex (Male *vs* female)	0.93 (0.64–1.35)	0.700	1.04 (0.72–1.49)	0.846	–		–	
Tumor location (Antrum *vs* non-antrum)	0.76 (0.53–1.09)	0.131	0.94 (0.67–1.33)	0.737	0.65 (0.45–0.96)	0.031	–	
Histology grade (III *vs* I/II)	1.51 (1.02–2.23)	0.038	1.45 (0.99–2.13)	0.053	1.52 (1.00–2.30)	0.050	1.30 (0.88–1.93)	0.188
TNM stage (III *vs* IB/II)	1.89 (1.28–2.79)	0.001	1.62 (1.13–2.33)	0.009	2.06 (1.37–3.10)	<0.001	1.84 (1.26–2.69)	0.002
MSI status (MSI *vs* MSS)	1.24 (0.68–2.25)	0.481	1.02 (0.55–1.90)	0.944	–		–	
Postoperative CT/RCT (Treated *vs* untreated)	0.32 (0.22–0.47)	<0.001	0.33 (0.23–0.47)	<0.001	0.32 (0.21–0.48)	<0.001	0.31 (0.21–0.47)	<0.001

*Variables were adopted for their prognostic significance (P < 0.15) by univariate analysis.

HR, hazard ratio; CI, confidence interval; MSI, microsatellite instability; MSS, microsatellite stability; CT, chemotherapy; RCT, radiochemotherapy.

### Tumor Microenvironment Status and Postoperative Chemotherapy/Radiochemotherapy Efficacy by Stages

**Figure 3:**
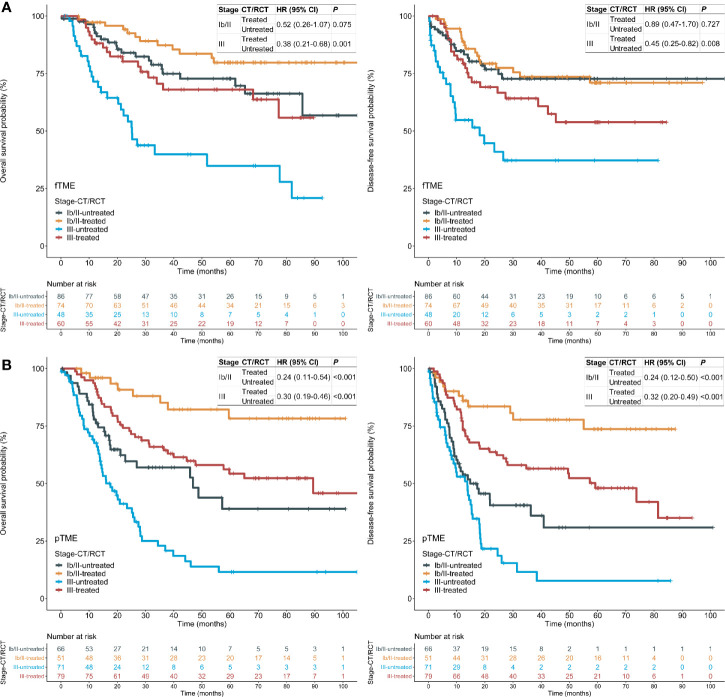
Tumor microenvironment (TME) status and the efficacy of postoperative chemotherapy (CT) or radiochemotherapy (RCT), stratified by stages in the pooled cohort. **(A)** fTME subset; **(B)** pTME subset. HR: hazard ratio; CI: confidence interval; fTME or pTME: favorable or poor TME.

As patient selection for postoperative CT/RCT is currently based predominantly on pathological staging, we performed stratified analyses using TNM staging. We found that the OS and DFS benefits of postoperative CT/RCT in fTME GC were limited to patients with stage III disease; these benefits in pTME GC were pronounced in both stage Ib/II and III disease (**Figure 3**), indicating that fTME may predict a lack of postoperative CT/RCT efficacy in stage Ib/II GC. Besides, patients with pTME GC benefited more evidently from postoperative CT/RCT than patients with fTME GC, even in cases with stage III disease (**Figure 3**).

### Tumor Microenvironment Status and Hypoxia

**Figure 4:**
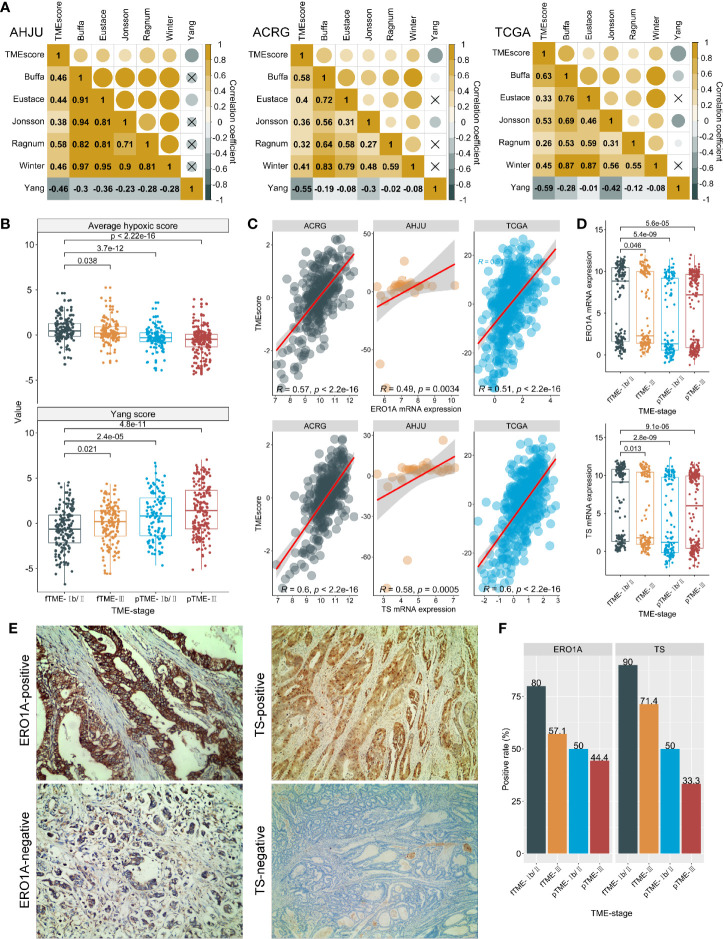
Tumor microenvironment (TME) status and hypoxia. **(A)**: Correlation between TMEscore and hypoxia scores (Authors who develop hypoxia signatures are shown); **(B)**: Average hypoxia score and RCT response score developed by Yang, stratified by TME status and stage. **(C)**: Correlations between TMEscore and ERO1A and thymidylate synthase (TS) mRNA expression. **(D)**: ERO1A and TS mRNA expression, stratified by TME status and stage; **(E)**: Typical micrographs for immunohistochemistry staining of ERO1A and TS proteins, at 200× magnification; **(F)**: Protein expressions of ERO1A and TS, by TME status and stage. AHJU, Affiliated Hospital of Jiangsu University; ACRG, Asian Cancer Research Group; TCGA, The Cancer Genome Atlas; fTME or pTME, favorable or poor TME.

As tumor hypoxia induces CT and RCT resistance (27), its association with TME status was analyzed. Surprisingly, TMEscore was positively correlated with the hypoxia scores (**Figure 4A**), suggesting that fTME is related to a greater degree of hypoxia than pTME. On the contrary, the TMEscore was negatively correlated with an RCT response score in association with hypoxia (19) (**Figure 4A**). Moreover, stage Ib/II fTME GC showed the highest mean hypoxia score and lowest RCT response score across all the subgroups in the pooled cohort (**Figure 4B**).

We further validated our findings through a novel endogenous hypoxia marker—ERO1A (28) and observed that the TMEscore was positively correlated with the mRNA expression of ERO1A in all the cohorts (**Figure 4C**). Similar results were found for the mRNA expression of TS (**Figure 4C**)—the target of FU and an indicator of poor CT outcomes—potentially in association with upregulation by hypoxia (21, 29). In addition, stage Ib/II fTME GC was associated with the strongest ERO1A and TS mRNA expressions across all the subgroups in the pooled cohort (**Figure 4D**).

Further validations were performed using IHC analysis in the AHJU cohort (**Figure 4E**). We found that the positive rates of ERO1A (12/17 *vs* 8/17; P = 0.163) and TS (14/17 *vs* 7/17; P = 0.013) were higher in fTME than pTME. Besides, stage Ib/II fTME GC showed the highest positivity rate for both ERO1A and TS proteins across all the subgroups (**Figure 4F**).

## Discussion

The treatment for GC has long been suboptimal, owing to a lack of validated prognostic or predictive biomarkers for therapy strategy optimization. The potential application of immune-associated biomarkers in resected cancers has recently been highlighted. Of them, tumor mutation burden (TMB) is a predictor of response to immune checkpoint inhibitors (30). A retrospective study on resected non-small-cell lung cancer found that a high TMB is correlated with favorable prognoses; however, a pronounced benefit from adjuvant CT was observed with a low TMB (31). In resected GC, MSI and PD-L1 expression, two other major predictors of immunotherapy efficacy, were found to be independent predictors of favorable prognoses; however, patients with MSI or stromal PD-L1-positive GC did not benefit from adjuvant CT (32). Recently, we also reported that MSI may predict poor response to postoperative RCT in patients with stage Ib/II GC (13). Based on these findings, we sought to investigate the association between TME status and the efficacy of postoperative CT/RCT in resected GC.

This study revealed that patients with pTME GC may benefit to a greater degree from postoperative CT/RCT than those with fTME GC. Specifically, the postoperative CT/RCT benefit was observed regardless of the disease stage in patients with pTME GC, but was limited in stage III fTME GC. These findings indicated that GC patients who are considered for postoperative CT/RCT should have the tumor TME status assessed to inform the likelihood of therapy benefit, which may help improve the outcome of postoperative CT/RCT.

Immune-associated biomarkers are reflective of tumor-host immune interactions (33, 34), partly explaining their clinical relevance in resected tumors. In this study, we validated the positive correlation between TME status and antitumor immunity using both mRNA-based bioinformatics and IHC-based assessment. Specifically, we found that fTME GC was associated with a high level of NK cell infiltration, consistent with the increasing body of evidence on the interaction between TME and NK cells (35, 36). However, fTME GC was related to a lack of CD8+ T cell infiltration. This result may be unsurprising considering the positive correlation between CD8+ T cell infiltration and the key immunosuppressive indicator—PD-L1-positive expression, as previously reported (37, 38) and also indicated in our study. These findings suggest that TME status, as evaluated by the TMEscore, is a specific biomarker of immune activation.

Previously, we reported that stage Ib/II MSI GC was the only subgroup that did not experience the benefits of postoperative RCT (13). In this study, a similar result was found for stage Ib/II fTME GC. Because MSI also correlated with more inflamed tumors, these findings indicate that dynamic immune infiltration may impair CT/RCT response. Similarly, in bladder cancer, a study found that immunotypes characterized by low rather than high levels of immune infiltration derive benefits from adjuvant CT (39).

Hypoxia can determine CT/RCT response (27). Correspondingly, we further revealed that fTME was more hypoxic than pTME. Specifically, stage Ib/II fTME GC showed the highest level of hypoxia, accompanied by the highest TS expression. Interestingly, active immune infiltration in fTME seemed to increase hypoxia level. Recently, elevated hypoxia was also found to be associated with increased TMB across cancer types including GC (40). Because high TMB promotes immune infiltration by producing more novel peptide epitopes or neoantigens (41), this finding indicated again the association between immune infiltration and hypoxia. More studies need to investigate this association.

This study has some limitations. First, we retrieved the patient data from public databases; some important information, including that on the CT/RCT regimens, criteria for CT/RCT decisions, surgical style, and margin status was incomplete and even missing, impacting our results. Second, the patients were not randomly selected in this retrospective study, highlighting the need for randomized prospective validations. Moreover, heterogeneity existed among the study populations in terms of the patients’ characteristics, NGS methods, and data processing methods, among others. However, we obtained consistent results among the three independent cohorts, indicating the robustness of our findings.

In conclusion, our results indicate that TME status is correlated with the efficacy of postoperative CT/RCT in resected GC and that fTME may predict a lack of postoperative CT/RCT response in stage Ib/II GC. Therefore, TME evaluation, especially in the setting of stage Ib/II GC, should be considered a clinically useful marker to identify patients who may fail in postoperative CT/RCT, and represents an additional step in individualized GC therapy. Further validations of our findings especially in randomized prospective studies are necessary.

## Data Availability Statement

The datasets presented in this study can be found in online repositories. The names of the repository/repositories and accession number(s) can be found in the article/**Supplementary Material**.

## Ethics Statement

The studies involving human participants were reviewed and approved by the Ethics Committee of the Affiliated Hospital of Jiangsu University. The patients/participants provided their written informed consent to participate in this study.

## Author Contributions

RD, XL, and DW conceived and designed the study. RD, DZe, XC, BS, DZh, LZ, YY, XL, and DW developed the methodology, RD, DZe, XC, BS, DZh, LZ, YY, XL, and DW acquired the data. RD, DZe, XC, BS, DZh, LZ, YY, XL, and DW analyzed and interpreted the data. RD, XL, and DW wrote, reviewed, and/or revised the manuscript. RD, DZe, XC, BS, DZh, LZ, YY, XL, and DW provided administrative, technical, or material support. RD, XL, and DW supervised the study. All authors contributed to the article and approved the submitted version.

## Funding

This project was supported by grants from the National Natural Science Foundation of China (81502130 and 81972822), Science and Technology Planning Social Development Project of Zhenjiang City (SH2019046), Project of Young Medical Talents in Jiangsu Province (QNRC2016829), 5123 Scholar Program of the Affiliated Hospital of Jiangsu University (51232017301), and Medical Science Research Fund from Beijing Medical and Health Foundation (YWJKJJHKYJJ-F2020E).

## Conflict of Interest

DZh and LZ are employees of Geneseeq Technology Inc. YY is an employee of 3D Medicines Inc.

The remaining authors declare that the research was conducted in the absence of any commercial or financial relationships that could be construed as a potential conflict of interest.

## Supplementary Material

Supplementary Figure 1 The efficacy of postoperative chemotherapy (CT) or radiochemotherapy (RCT) in resected gastric cancer. AHJU, Affiliated Hospital of Jiangsu University; ACRG, Asian Cancer Research Group; TCGA, The Cancer Genome Atlas; HR, hazard ratio; CI, confidence interval.
