# Supplementary figures and images for: Tumor Microenvironment Status Predicts the Efficacy of Postoperative Chemotherapy or Radiochemotherapy in Resected Gastric Cancer
Source: Front Immunol. 2021 Jan 25;11:609337. doi: 10.3389/fimmu.2020.609337 (PMC7868549; doi:10.3389/fimmu.2020.609337)

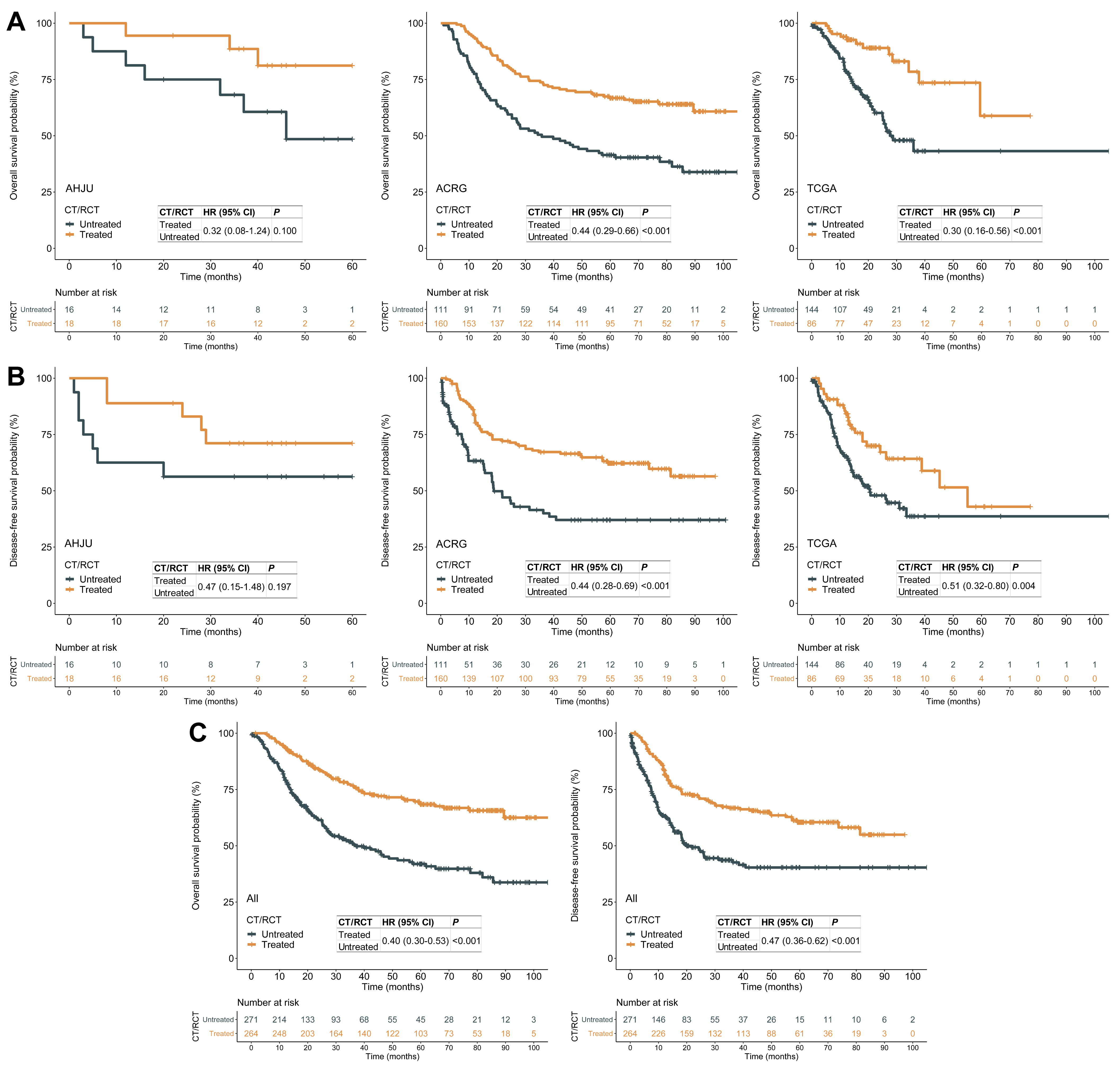

Supplement: Supplementary Figure 1 — The efficacy of postoperative chemotherapy (CT) or radiochemotherapy (RCT) in resected gastric cancer. AHJU, Affiliated Hospital of Jiangsu University; ACRG, Asian Cancer Research Group; TCGA, The Cancer Genome Atlas; HR, hazard ratio; CI, confidence interval. [file Image_1.tif]
